# Comments on ‘Comparison of anticoagulation strategies for veno-venous ECMO support in acute respiratory failure’: the bitter truth about unfractionated heparin monitoring assays

**DOI:** 10.1186/s13054-021-03525-5

**Published:** 2021-03-29

**Authors:** Mouhamed Djahoum Moussa, Osama Abou-Arab, Emmanuel Robin, André Vincentelli

**Affiliations:** 1 CHU Lille, Pôle d’Anesthésie-Réanimation, F-59000 Lille, France; 2 Department of Anesthesiology and Critical Care Medicine, Amiens University Hospital, Amiens, France; 3 CHU Lille, Service de Chirurgie Cardiaque, F-59000 Lille, France; 4 Service d’Anesthésie-Réanimation Cardiovasculaire et Thoracique, Institut Cœur - Poumon, CHU Lille, 2 avenue Oscar Lambret, 59 037 Lille, France

**To the editor**,

We read with interest the article by Benjamin Seeliger et al. [1] in which the authors compared two different anticoagulation strategies defined as high-dose (HD) heparinization and low-dose (LD) heparinization. They report a lower rate of ECMO oxygenator change and thromboembolic events, in the HD group as compared to LD group.

1. The limits of activated clotting time (ACT) and partial thromboplastin time (PTT) to monitor ECMO anticoagulation.

LD and HD heparinization were defined using different anticoagulation monitoring assays—PTT and ACT respectively—leading to a possible classification bias. These assays are known to be poorly correlated to heparin concentration, to heparin anti-FXa and to each other [2].

Notably, the mean PTT values in the HD and LD groups [48 s (IQR 41–57) vs 38 s (IQR 34–42)] were overlapped for 25% of patients despite a significant difference in heparin dose. This underlines the poor correlation between heparin concentration and the monitoring assays used. Moreover, the difference in mean PTT is uninterpretable without providing for each PTT assay, the heparin therapeutic range is corresponding to the anti-FXa range of 0.3–0.7 UI/ml [3].

Furthermore, the accuracy of PTT and even worse of ACT are sensitive to several analytical limitations, and biological factors (thrombocytopenia, coagulation factor deficiencies, etc.) unrelated to heparin therapy are commonly observed during ECMO support [2, 4].

In addition, the ELSO recommends that ACT or PTT should not be used in isolation for heparin monitoring due to their limitations [5].

2. The observed differences in thrombotic events cannot be explained by the difference in heparin dosage alone.

The transfusion strategies in both centers differed significantly and should not be ignored in the analyses. Patients in the LD heparinization group received more platelet concentrates and more prothrombin complex concentrates, in line with the liberal transfusion practices in this center. However, these confounders were not included in the multivariate analysis.

Since the baseline coagulation covariates (fibrinogen, d-dimers, antithrombin) included in the model are likely to fluctuate daily during ECMO, they should be analyzed as time-varying covariates in the multivariate model. ACT and PTT may have similar variations, so it would be more informative to analyse them as time-dependent covariates in a multivariate model to explain thrombotic and bleeding complications.

In conclusion, this study has compared the ECMO management procedures of two centers beyond heparinization alone, in two different populations with a methodology that may lead to misinterpretation.

## Data Availability

Not applicable.

## Abbreviations

Anti-FXa: Antifactor Xa
ECMO: Extracorporeal membrane oxygenation
ELSO: Extracorporeal life support organization
IQR: Interquartile range
