# Supplementary material for: Comments on ‘Comparison of anticoagulation strategies for veno-venous ECMO support in acute respiratory failure’: the bitter truth about unfractionated heparin monitoring assays
Source: Crit Care. 2021 Mar 29;25:124. doi: 10.1186/s13054-021-03525-5 (PMC8006337; doi:10.1186/s13054-021-03525-5)
Supplement: Related texts

# Reply: Authors’ response

**Authors:** Benjamin Seeliger, Andreas Tiede, Sascha David, Christian Bode

**Affiliations:** 5 grid.10423.34 0000 0000 9529 9877 Department of Respiratory Medicine, Hannover Medical School and German Centre for Lung Research (DZL), Hannover, Germany; 6 grid.10423.34 0000 0000 9529 9877 Department of Haematology, Haemostasis, Oncology, and Stem Cell Transplantation, Hannover Medical School, Hannover, Germany; 7 grid.412004.3 0000 0004 0478 9977 Institute of Intensive Care Medicine, University Hospital Zurich, Zurich, Switzerland; 8 grid.15090.3d 0000 0000 8786 803X Department of Anaesthesiology and Intensive Care Medicine, University Hospital Bonn, Bonn, Germany

**To the editor**,

We thank *Moussa *et al*.* for their comments on our article, addressing the issue of heparin monitoring and potential confounders.

Concerning the monitoring of heparin, we agree that APTT and ACT poorly correlate with each other and with anti-Xa levels in critically ill patients. Our own group recently confirmed this observation using two modern ACT devices with different detection methods [6]. As outlined in the current paper, we employed additional anti-Xa measurements whenever APTT or ACT was inconsistent with heparin dose or clinical picture. The main study result was a significant difference in heparin dose (17,495 vs 11,185 IU/d, *p* < 0.001) and a remarkable difference in the risk of oxygenator change between the two centers.

An intriguing question is why most centers, despite ELSO recommendations and the well-known limitations of APTT/ACT, still use these assays in the majority of patients [7]. Is it ignorance and lethargy that anti-Xa assays have not been adopted more completely? Or is it their longer turnaround time, higher cost and lack of 24/7 availability? It must be acknowledged that anti-Xa assays do not measure heparin *concentration* but just one of its pharmacodynamic, antithrombin-dependent *effects*. Different anti-Xa assays (with or without addition of exogenous antithrombin) do not measure the same in critically ill patients with low antithrombin [8]. Neither do anti-Xa assays reflect the important effects heparin has on other coagulation enzymes and the expression of tissue factor and tissue factor pathway inhibitor [9]. Proof is lacking that anti-Xa monitoring improves the outcome of ECMO patients. So-called global hemostasis assays may possibly better reflect the complex alterations of hemostasis and guide the use of heparin and blood components [10].

Concerning the transfusion strategy as a potential confounder, we agree with *Moussa *et al. and concluded already in our paper that prospective studies with predefined transfusion strategies are needed. We do not agree that transfusions could have been simply included as covariates in our analysis. The dilemma is that transfusion, for example, of platelets, may potentially prevent bleeding but is also done because of already existing bleeding.

In conclusion, our retrospective comparison of real-world data from two centers found lower rates of oxygenator change and thromboembolic complications in the center employing higher doses of heparin. Patient baseline characteristics were unlikely to explain this difference. Because of other potential confounders (e.g. transfusion strategy), confirmation from prospective studies is required.
